# Light-driven high-precision cell adhesion kinetics

**DOI:** 10.1038/s41377-022-00963-w

**Published:** 2022-09-13

**Authors:** Zhiyuan Zhang, Daniel Ahmed

**Affiliations:** Acoustic Robotics Systems Laboratory, Institute of Robotics and Intelligent Systems, Department of Mechanical and Process Engineering, ETH Zurich, Säumerstrasse 4, CH-8803 Zurich, Switzerland

**Keywords:** Optical manipulation and tweezers, Micro-optics

## Abstract

Existing single-cell adhesion kinetics methods are performed under conditions highly unlike the physiological cell adhesion conditions. Now, researchers have developed a new optical technique for high-precision measurement of cell lateral adhesion kinetics in complex clinical samples.

Cell adhesion is highly involved in many biological processes, such as cell communication^1^, tissue development^2,3^, virus invasion^4,5^, and cancer metastasis^6,7^. A variety of single-cell adhesion methods have been developed based on atomic force microscopy (AFM)^8–10^, optical tweezers^11–13^, magnetic tweezers^14–16^, acoustic tweezers^17–19^, micro-needle manipulation^20^, and biomembrane force probes^21^. These methods all depend on repeatedly rupturing the adhesive contact in the normal direction of the cell interface so as to measure the normal tensile force^22,23^; however, different methods can still yield measurement results that differ by several orders of magnitude. These discrepancies stem from dynamic changes in sample interactions and a lack of consideration for lateral adhesion kinetics^24^.

Preliminary studies have shown that in vivo cell adhesion under physiological flow is more complex, and that lateral adhesion kinetics play a significant role in the dynamic modulation to withstand changing flow^25–28^. Currently, there exist microfluidic techniques for analyzing the lateral force along the tangential direction^29–31^; however, these methods face limitations in terms of time required for measurement, cell interaction distance, and measurement resolution. High-precision and high-speed in situ measurement of lateral adhesion kinetics remains an open challenge.

In this issue of *eLight*, Yuebing Zheng’s research team at the University of Texas at Austin in USA presents a new optical technique, termed the single-cell rotational adhesion frequency assay (scRAFA). This method integrates optical trapping, rotation, imaging, and spectroscopy on a single platform. ScRAFA exploits a microfluidic platform integrated with versatile optothermal manipulation and optical imaging capabilities to stably trap and rotate any specific single cell, continuously monitor the complete cell adhesion process from initiation of bonding with the substrate to formation of permanent attachment (response time <0.1 s), and precisely control the interaction distance between substrate ligands and cell receptors (resolution ±0.1 nm), which control cannot be achieved in a conventional flow chamber assay^32^. More specifically, a focused 785 nm laser beam was first applied to trap the cell with optical force, then the temperature gradient field produced by a focused 532 nm laser beam was used to rotate the cell. Subsequently, Zheng’s team retrieved the time-dependent light intensity signals from collected images to quantify the cell’s adhesion behaviors. By testing the lateral cellular interactions caused by flow-induced shear stress, they were able to successfully measure the adhesion strength of yeast cells in human urine and obtain more accurate dissociation constants, which is ~10 times more accurate than previous measurements.

Different from many of the existing adhesion measurement methods, the proposed light-driven scRAFA can reveal the shear-force-dependent adhesion behaviors of individual cells while in physiological fluids with various surface conditions. With its superior performance and general applicability, scRAFA will be a valuable tool in a wide range of fields, from cell biology to immunotherapy, biomedicine, and engineering.
